# Outbreak of Post‐Infectious Bronchiolitis Obliterans (PIBO) After Adenovirus Infection: A Case Series and Review of the Literature

**DOI:** 10.1002/ppul.71080

**Published:** 2025-04-01

**Authors:** A. Traunero, S. Ghirardo, M. Aldeco, P. Pascolo, S. Basilicata, L. Mazzari, M. Maschio, A. Amaddeo, U. Krivec

**Affiliations:** ^1^ Department of Medical Surgical and Health Sciences, University of Trieste Trieste Italy; ^2^ Department of Paediatric Pulmonology University Medical Centre Ljubljana, University Children's Hospital Ljubljana Ljubljana Slovenia; ^3^ Department of Translational Medical Sciences, Paediatric Pulmonology Federico II University Naples Italy; ^4^ Institute for Maternal and Child Health IRCCS “Burlo Garofolo” Trieste Italy

**Keywords:** adenovirus, bronchiolitis obliterans, diffuse lung disease, post‐infectious bronchiolitis obliterans

## Abstract

**Background:**

Post‐infectious bronchiolitis obliterans (PIBO) is a rare chronic pediatric pulmonary disease characterized by irreversible fibrotic narrowing of the small airways. Treatment options remain uncertain with limited success.

**Objective:**

To delineate the characteristics of patients diagnosed with PIBO in Ljubljana (Slovenia) and Trieste (Italy) in 2023.

**Methods:**

We retrospectively assessed clinical records of PIBO patients from January to December 2023, capturing data on initial viral infection, clinical presentation, radiological features, treatments, and outcomes.

**Results:**

In 2023, 11 patients were identified, contrasting with only 6 cases in the previous 7 years. Common symptoms and signs included tachypnea, chronic wet cough, and diffuse crackles following adenovirus pneumonia. Most patients were previously healthy. Chest CT findings confirmed the diagnosis in all cases. Bronchoalveolar lavage showed elevated levels of neutrophils (46% to 90% of cells), and biopsies performed in 6 patients indicated predominantly lymphocytic inflammatory infiltrate and bronchiolar fibrosis. Nocturnal pulse oximetry revealed reduced mean SpO2 (median: 96.5% Q1: 93%, Q3: 98%) and reduced lower values (median: SpO2 89% Q1: 87%, Q3: 92.5%) with an increased oxygen desaturation index (1.1 to 11.2 events/hour). Treatment involved methylprednisolone (20–30 mg/kg) for three consecutive days monthly for 6 months, resulting in clinical improvement in nine patients and radiological improvement in seven patients.

**Conclusions:**

The post‐pandemic surge in PIBO cases may stem from viral ecology changes, immunologic factors, and/or adenovirus genotypes, highlighting the need for further research into its etiology and management strategies.

## 1 Introduction

Bronchiolitis obliterans (BO) is a rare chronic obstructive pulmonary disease characterized by irreversible obstruction of the small airways. Lung and hematopoietic stem cell transplantation are the two leading causes of BO in adults [1, 2, 3, 4]. Additionally, BO can result from rheumatologic diseases or inhalation of toxic compounds such as nitrogen and sulfur dioxide, dust, and ash [5].

Post‐infectious BO (PIBO) is the most frequent cause of BO in childhood. The most common pathogens are adenovirus, Mycoplasma pneumonia, measles, influenza, or respiratory syncytial virus. After an acute viral or bacterial injury to the bronchiolar epithelium, a subepithelial inflammatory response leads to progressive parietal fibrosis and intraluminal bronchiolar obliteration [1]. The immunological mechanisms are still unknown; however, lung biopsies and bronchoalveolar lavage (BAL) fluid often show chronic neutrophilic infiltrates [6]. Unresolved inflammation leads to granulation tissue proliferation causing nonuniform fibrotic narrowing and obliteration of the bronchioles [7].

The diagnosis of PIBO is challenging since this condition is scarcely symptomatic until advanced stages, and its presentation resembles other more common airway diseases, such as asthma. Treatments range from nothing to, intravenous immunoglobulins, to azithromycin alone or in combination with fluticasone and montelukast, to methylprednisolone pulse therapy, but neither the duration of therapy nor the number of cycles is known. This study aimed to describe an adenovirus‐associated PIBO outbreak in our region during 2023. We aimed to analyze a cohort of pediatric patients affected by PIBO, including the treatments administered and clinical outcomes.

## 2 Methods

We conducted a retrospective study on recorded data of pediatric patients at two different academic medical centers, the University Children's Hospital of Ljubljana, Slovenia, and the Institute for Maternal and Child Health‐IRCCS Burlo Garofolo, in Trieste, Italy. All patients diagnosed with PIBO from January 01, 2023 to December 31, 2023 were included. At both centers, the diagnosis of PIBO was performed according to Yazan and Colom criteria, thus we excluded other chronic pulmonary conditions such as cystic fibrosis, motile ciliopathies, and immunodeficiencies [8, 9].

For each patient diagnosed from January 01, 2023 to December 31, 2023, we gathered the following information: birth date, sex, date of the pulmonary infection presumed to trigger PIBO, infection severity in terms of the need for respiratory support and its kind, the number of days spent on respiratory support, length of stay, access to intensive care unit (ICU), infective agent isolated using multiplex PCR test conducted on swab or nasopharyngeal aspirate, and need for respiratory support during the infective phase. We recorded all the symptoms and clinical signs reported in the clinical chart at the time of the diagnosis of PIBO, the date of diagnosis, high‐resolution computed tomography (HRCT) scan date and features. We also registered the type and duration of respiratory support used after the PIBO diagnosis, the treatment received (including duration and dosage), and any radiological and clinical changes observed after treatment such as symptoms reported by parents and children, need for antibiotics courses and physical examination. We recorded symptoms as reported in the clinical charts: cough improvements, wheezing improvements and the general parental feeling about their child's health state. Furthermore, we recorded data about bronchoscopy, lung biopsy, sleep studies, and pulmonary function tests.

### 2.1 Statistical Analysis

Dichotomous variables were reported as numbers and percentages, continuous variables as mean and standard deviation when normally distributed and median plus first and third quartile when non‐normally distributed. The normality of data was graphically assessed and verified using the Shapiro–Wilk test. To evaluate diagnostic latency, we used the Log‐rank test. We calculated the 95% confidence interval of the difference, expressed in days between the two groups.

## 3 Results

### 3.1 Patients' Data and Clinical History

From January 01, 2016 to December 31, 2022, we diagnosed a total of 6 patients as having PIBO, less than one case per year; in every patient adenovirus was identified as the putative causative agent, the median age at diagnosis was 3.3 years (range: 2.3–4.3). From January 01, to December 31, 2023, we diagnosed 11 children with PIBO (seven patients from University Children's Hospital and four patients from Burlo Garofolo Hospital), eight boys and three girls. In 2023, the median age at diagnosis was 2.6 years (range: 1.3–6.3 years).

All 11 patients had been previously affected by moderate to severe viral lower respiratory tract infections, requiring ICU care in three cases. Ten patients (91%) needed respiratory support, ranging from low‐flow oxygen therapy (*n* = 4) and high‐flow nasal cannulas (HFNC) (*n* = 5) to noninvasive ventilation (NIV) in one case, followed by continuous positive airway pressure support (CPAP) for 40 days. No one required invasive mechanical ventilation. Patient 4 was treated with NIV and subsequently CPAP. Patient 4 has Down syndrome, while the remaining ten patients had an unremarkable previous medical history. All except patient 4 were discharged from hospital after the infective acute phase before the diagnosis of PIBO.

During the acute phase, as part of the standard of care for patients with acute respiratory failure and most of the patients admitted for respiratory infections, all patients were tested using polymerase chain reaction (PCR) viral panels of the 13 common viral respiratory agents, including RSV, SARS‐CoV‐2, Influenza virus, mycoplasma and Rhinovirus (Respiratory Flow Chip assay—Vitro, Sevilla, Spain). Adenovirus was detected in all 11 patients and was the sole pathogen identified in 6 cases. Of the remaining instances (*n* = 5) one was coinfected with parainfluenza virus, one with bocavirus, and three with respiratory syncytial virus, one of whom also tested positive for rhinovirus. Patients' characteristics during the infection by adenovirus are reported in Table 1. 6 patients required HFNC, 4 patients low flow oxygen, 1 patient NIV and CPAP; the average number of days for respiratory support was 9.9, median 6, Q1 5, Q3 10.5. In most cases, tachypnea (four patients), cough (10 patients), exercise intolerance (four patients), wheezing (one patient) and other nonspecific symptoms began manifesting with a median interval of 1 month (range: 0–5 months) after acute infective phase recovery. Chronic cough, was the most frequently reported symptom, followed by exercise exacerbated cough. The cough was mostly described as wet by the parents in all but one of our cases.

**Table 1:** Patients' characteristics during adenovirus infection.

	age (months)	sex	ICU	Respiratory support type and its duration (days)	Coinfections	Symptoms at PIBO diagnosis	Signs of PIBO at diagnosis	Drugs at PIBO diagnosis
pts 1	13	M		HFNC, 5	Rhino, RSV	C	Cr	No treatment
pts 2	12	M		HFNC, 6		T, C	W, Cr	azithromycin (100 mg x 3/week); methilprednisolone 1 bolus
pts 3	29	M		O2, 4		T, C	W	azithromycin 3.5 ml (140 mg x 3/week)
pts 4	67	F	x	HFNC, CPAP, NIV, 40		T, C, E	H, Cr, Hy, Cwr	CPAP + MgSO4 + methylprednisolone
pts 5	26	M	x	HFNC, 5	RSV	T	Cr	No treatment
pts 6	23	F		O2, 6		E	Cr, Cwr	Fluticasone 125 mcg BID
pts 7	17	F		O2, 10	RSV	C, E	Cwr, B	No treatment
pts 8	16	M		O2, 6	Boca	E	Cr, Cwr	Fluticasone 125 mcg BID
pts 9	15	M		HFNC, 12		W	Cr	No treatment
pts 10	14	M	x	HFNC, 5	Para	C		Fluticasone 50 mcg BID
pts 11	70	M		/		C	Cr	amoxicillin‐clavulanate for 27 days, not beneficial;
Median; Q1‐Q3	20; 18 ‐ 31			6; 5‐10,5				

Abbreviations: B, bronchial breath sound; BID, Bis In Die; Boca, bocavirus; C, Cough; CPAP, continuous positive airway pressure; Cr, crackles; Cwr, chest wall retractions; E, exercise intolerance; H, hypoxemia; HFNC, High Flow Nasal Cannulae; Hy, hyperinflation, NIV, noninvasive ventilation; Para, parainfluenza virus; Rhino, rhinovirus; RSV, respiratory syncytial virus, T, tachypnoea; W, wheezing.

In 8 patients crackles were heard, diffusely or predominantly at the chest base (mono‐ or bilaterally), four presented chest/intercostal retractions, and three wheezing. We detected bronchial breathing along the right hemithorax in one child who had a complete atelectasis of the right lung. The child who required NIV and CPAP support during the lower respiratory tract infection demonstrated persistent low‐flow oxygen dependence to maintain target saturations and avoid dyspnea and also presented signs of thorax hyperinflation. At diagnosis patients presented a median weight percentile of 54 (Q1: 20, Q3: 64), a length percentile median 63 (Q1: 20, Q3: 94).

### 3.2 Diagnostic Workup

A median of 123 days passed between the viral infection and PIBO diagnosis (Q1: 59, Q3: 177 days). This time span significantly increased, with a median of 62 days (Q1: 42, Q3: 107) from January 01 to June 30 2023, and a median of 177 days (Q1: 148, Q3: 211) from July 01 to December 31, 2023 (*p* < 0.0001). The time interval between adenovirus infection and PIBO diagnosis in the historical cohort (January 1, 2016–December 31, 2022) had a median of 7.5 months (range: 2.8–37.3). Although this interval was lower in 2023, the difference was not statistically significant (*p* = 0.22).

HRCT was performed on all patients, revealing air‐trapping zones in all subjects (*n* = 11), characteristic mosaic perfusion (*n* = 10) as consequence of vascular attenuation and air trapping, thickened bronchial walls (*n* = 9), and atelectasis (*n* = 9). Additionally, mucoid bronchial impaction (*n* = 4), consolidations (*n* = 2), and bronchiectasis (*n* = 1) were found in the HRCT scans. Air trapping, mosaic perfusion, bronchial thickening and atelectasis were recognized in almost all patients (in 100%, 91%, 82% and 82% of the patients respectively), which is very similar to the literature.

We did not perform any swallow test in our patients, except for the girl with Down syndrome who underwent a dedicated swallow clinical evaluation and barium contrast swallow test. Both tests yielded normal results; therefore, no feeding modifications were made. For the other patients, we relied solely on parental reports regarding episodes of choking or coughing during meals, all of which were denied.

All patients underwent a sweat test and/or genetic testing, excluding cystic fibrosis. We checked immunoglobulins levels, lymphocyte subpopulation count, protein electrophoresis, complement factors assay including the evaluation of the lectin pathway of the complement system, and immune responses to vaccines. Primary ciliary dyskinesia (PCD) was ruled out due to clinical history and genetic testing was performed in four patients. Five patients underwent genetic whole exome analysis searching for childhood interstitial lung diseases (chILD), while another underwent testing for immunodeficiencies. All genetic results were found to be normal.

Eight patients underwent bronchoscopy after the diagnosis of PIBO but before administration of the first bolus of methylprednisolone showing no anatomical or malacic anomalies. BAL was performed in all 8 patients instilling 3 mL per kg of body weight divided in three equal aliquots, revealing a remarkable neutrophilia, ranging from 46% to 90%. Haemophilus influenzae was detected in six patients as the single agent or in conjunction with other pathogens. Other respiratory bacteria were identified in individual patients, including Pseudomonas aeruginosa in the patient with bronchiectasis and Down syndrome, Streptococcus pneumoniae, Streptococcus pyogenes, and Moraxella catarrhalis. Among viruses, rhinovirus, bocavirus, and parainfluenza virus were identified in three different patients, while adenovirus was detected in only one child. Furthermore, Pneumocystis jirovecii and *Candida albicans* were detected in association with viral and bacterial agents.

We performed lung biopsy in 6 patients through transbronchial biopsy (TBB) (*n* = 4), video thoracoscopic assisted surgery (VATS) (*n* = 1) or right thoracotomy (*n* = 1). The histological findings were sparse (see Table S1 for the results of every biopsy).

We conducted the multiple‐breath washout test using sulfur hexafluoride (MBWSF_6_) to assess lung function measuring lung clearance index (LCI 2.5%) in 5 patients, revealing a median LCI of 14.5 (Q1: 10.5, Q3: 18.9; minimum 10.4, normal value < 7) reflecting ventilation inhomogeneity. Functional residual capacity (FRC) measurements were performed in five patients, resulting in a median value of 18 mL/kg (Q1: 15, Q3: 51.6). Tidal volume was measured in six patients, showing a median of 11.5 mL/kg (Q1: 10.5, Q3: 13) with normal values of 8–15 mL/kg. Despite normal daytime peripheral oxygen saturation levels, six out of the eight children investigated through nocturnal pulse oximetry showed SpO2 levels below 95%. Specifically, four patients exhibited minimum SpO2 levels below 90%, with an oxygen desaturation index (ODI) showing wide variability (ranging from 1.1 to 11.2).

### 3.3 Therapies

At the acute infection clinical presentation, bronchodilators and steroids (in various combination) had already been attempted without consistent improvement. Three patients received an oral corticosteroid course at diagnosis of PIBO, followed by daily salmeterol/fluticasone and azithromycin (10 mg/kg/dose) three times per week. Four patients received salbutamol and intravenous methylprednisolone. Following PIBO diagnosis, all patients received corticosteroid pulse therapy with methylprednisolone (20–30 mg/kg/day up to 1 g) for 3 consecutive days every month for a maximum of six consecutive months. All but one kept taking azithromycin.

Other treatments included montelukast (*n* = 1) and intravenous immunoglobulins (IVIG) (*n* = 2). One of the two patients who received IVIG was subject 4, as she exhibited a reduced T‐memory cell repertoire. Considering the remarkably peribronchiolar and interstitial fibrotic features of her biopsy, nintedanib was started at a daily dose of 50 mg in two divided doses. Due to disease progression, the same patient received cyclophosphamide (500 mg/m^2 of body surface area) monthly for 6 months. Then she commenced oral mycophenolate mofetil daily (860 mg/m^2 divided into two doses).

Respiratory physiotherapy was implemented as an additional supportive measure for all compliant patients (*n* = 10) who underwent twice‐daily sessions with a PEEP valve (*n* = 7) or bottle‐positive expiratory pressure (PEP) (*n* = 3). We also used hypertonic saline inhalation twice daily before respiratory physiotherapy to improve mucociliary clearance (*n* = 7). Physiotherapy was started concomitantly with PIBO diagnosis in all the cases and perceived as helpful in mucous clearance by all but two families.

### 3.4 Outcomes

At the moment of definitive submission of the article the median follow‐up was of 476 days (Q1: 365, Q3: 525), 9 patients had clinically improved in terms of parent reported symptoms (cough frequency and quality, exercise tolerance and wheezing) and chest auscultation (wet sounds and wheezing). Seven required at least one cycle of intravenous antibiotics. Additionally, seven patients received one (*n* = 3) or more cycles (*n* = 4) of oral antibiotic therapy but did not require hospitalization.

One child developed secondary arterial hypertension occurred concomitantly to steroid boluses, requiring chronic therapy with a combination of antihypertensive drugs (amlodipine, enalapril). No other complications occurred among this cohort of patients.

Subject 4 was the only one needing long‐term oxygen therapy at home, switching from low to high flow after 3 months. After cyclophosphamide boluses it was possible to partially wean her off during the daytime.

**Figure 1:**
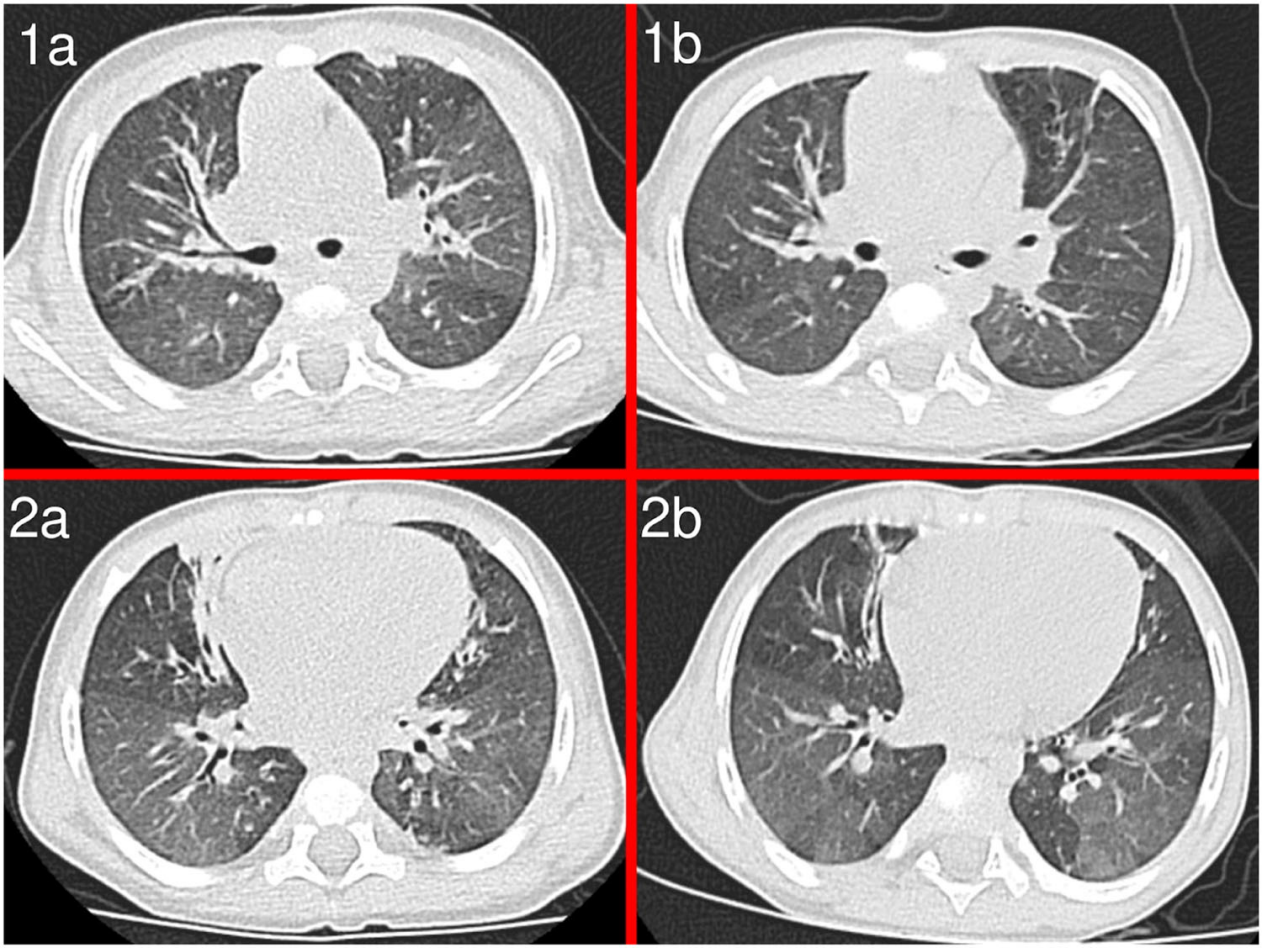
CT findings (patient 2). 1‐2a: CT scans before steroid treatment, showing peri‐bronchial inflammation, mosaic perfusion features and air trapping areas. 1‐2b: CT scans after steroid cycles, documenting a reduction of peribronchitis with unchanged mosaic perfusion pattern. [Color figure can be viewed at wileyonlinelibrary.com]

CT scans were performed within a month after the completion of steroid cycles in 9 patients. Improvement was seen in 7 patients, all of whom improved clinically as well. The improvement in peribronchitis was the most evident aspect, followed by emptying of bronchiectasis and healing of consolidation. The mosaic perfusion pattern did not change. Figure 1 shows HRCT features of two patients before and after the steroid boluses.

At the time of writing, follow‐up CT scans were still pending for two patients. In one case parents declined the follow‐up CT in one case and the patient with right lung atelectasis presented no improvement at the X‐ray, so a CT was not performed. Chest X‐rays were performed as part of the follow‐up in three children who did not undergo follow‐up CT scans, and improvement was observed in two of them.

Thus, 7 patients showed a radiological improvement.

One month after the completion of the steroid cycles, LCI improved in three of the four patients retested, with a median LCI 2.5% of 10.5 (Q1: 9.21, Q3: 11.85, minimum 9.2).

At the last evaluation patients presented a median weight percentile of 53 (Q1: 43, Q3: 94), a length percentile median 75 (Q1: 30, Q3: 95). At the last evaluation the median SpO2 was 96% (Q1: 96%, Q3: 97%); median RR was 24 (Q1: 24, Q3: 31); no patient presented chest retractions nor other signs of dyspnea.

Patient's characteristics, treatments and outcomes are summarized in Table 2

**Table 2:** Patients features and treatments for PIBO.

	Age at diagnosis	CT findings					BAL (bacteria, viruses & fungi)	Biopsy	Treatment (n. boluses, other treatments) diagnosis to first bolus	Improvement: Clinical/radiological
		Mosaic pattern	Air trapping	Bronchial wall thickening	Atelectasis	Other				
Pts1	20	X	X	X	X	M	Av, Hi	Ei, N, Ma	6, Azt; 8	no/yes
Pts2	16	X	X	X	X	C		\	6, Azt, Ig; 0	yes/yes
Pts3	30	X	X	X		B		\	6, Azt; 3	yes/yes
Pts4	67	X	X	X	X		Pa	Fi, Co, Li	5, Azt, Ig, Cyp, Nnt, My; 0	yes/yes
Pts5	31	X	X	X	X		Pv, Mc	\	6, Azt; 14	yes/no
Pts6	26	X	X	X		M	Hi, Sp	\	6, Azt; 14	yes/yes
Pts7	18	X	X		X	C	Rv, Cmv, Hi, Ca	Co, Li, Fi, Mo, N, Ma	6, Azt; 19	no/no
Pts8	18	X	X	X	X	M	Bv, Hi	Ei	4, Azt, Ml; 12	yes/no data
Pts9	19		X		X		Hi	Li, N	6; 72	yes/yes
Pts10	20	X	X	X	X	M	Hi, Spy, Mc, Rv, Pc	inappropriate sample	6, Azt; 9	yes/no
Pts11	75	X	x	X	x				6, Azt; 8	yes/yes
Median Q1‐Q3										

*Note:* The age at PIBO diagnosis is reported in months and the time between diagnosis to the first bolus is reported in days.

Abbreviations: Av, adenovirus; Azt, azithromycin; B, bronchiectasis; Bv, bocavirus; C, consolidation; Ca, *Candida albicans*; Co, collapsed alveoli; Cmv, citomegalovirus; Cyp, cyclophosphamide; Ei, eosinophilic infiltrate; Fi, fibrosis; Hi, Haemophilus influenzae; Ig, immunoglobulines; Li, lymphocytic infiltrate; M, mucoid bronchial impaction, Ma, macrophages; Mc, Moraxella catarrhalis; Ml, montelukast; Mo, mononuclear infiltrate; My, mycophenolate; N, neutrophils, Nnt, nintedanib; Pa, Pseudomonas aeruginosa; Pc, Pneumocystis jiroveci; Pv, parainfluenza virus; Rv, rhinovirus; Sp, Streptococcus pneumoniae; Spy, Streptococcus pyogenes.

Our historical cohort of 6 patients (from January 2016 to December 31, 2022) was diagnosed at an average age of 1.7 years, with a median of 1 year (Q1: 1 year, Q3: 3 years). At the last follow‐up evaluation, the cohort presented with a median tidal volume of 128 mL (Q1: 118, Q3: 132). The median FEV1 was 74% (Q1: 58%, Q3: 86%) and the median FVC was 89% (Q1: 50%, Q3: 96%). Three patients were taking chronic medications with ICS, and one of them was on azithromycin. None of the patients diagnosed in 2023 is currently able to perform spirometry at the time of writing.

## 4 Discussion and Literature Review

We are reporting an outbreak of PIBO characterized by an unprecedented increase in the number of cases in our region. Typically, PIBO is extremely rare, with limited descriptions in the literature and lacking a defined incidence rate. This literature aligns with our previous experience of fewer than one new case per year between 2015 and 2023, including 5 years before the onset of pandemic. Since this condition seems to be overlooked in milder cases [7] we might speculate about a few possible undetected mild cases during these years. On the other hand, as all our cases presented with moderate to severe involvement, it seems very unlikely that we would have missed a similar diagnosis in our setting.

We believe that adenovirus likely served as the triggering factor in every patient of our study as we identified it in each one. This is because adenovirus is the leading cause of PIBO, accounting for approximately 71% of cases of PIBO occurring in children under the age of 3 years [10], and the risk of developing PIBO following a lower respiratory infection caused by adenovirus is estimated to be roughly 28% [11]. The prevalence of male in our study may be of some importance for the understanding of the disease in light of sex specific respiratory literature [12].

We consider it likely that the substantial shifts in viral distribution resulting from the COVID‐19 pandemic and its containment measures had a significant impact. We can speculate that the risk of PIBO may have increased, possibly because of a combination of the following hypotheses. A different strain of adenovirus could have emerged through a genotype replacement mechanism like other viruses [13, 14, 15]. Unfortunately we were able to identify the adenovirus genotype in only one patient, who had an infection by type 3 virus [16, 17] Another potential explanation could be the increase in the number and severity of viral infections compared to the pre‐pandemic period [18]. The severity of the infection is known to correlate strictly with PIBO risk as PIBO develops more frequently if the patient requires mechanical ventilation, presents multifocal pneumonia, hypercapnia or a length of stay exceeding 30 days [11]. Moreover, the nearly complete absence of viral infections during the pandemic may have impaired immunological response to viruses [19], including a typical PIBO trigger such as adenovirus. The individual immunological response seems to be key in PIBO development, as even minor immunological defects, like a deficiency in mannose‐binding lectin (MBL) is more than twice as common among patients with PIBO [20].

As 9 out of 11 patients showed clinical improvement, we consider this outbreak to be less severe compared to the literature [20]. Although long‐term outcomes remain poorly defined some slow improvement of lung function during childhood is expected. Improvement is nearly exclusively limited to an increase of the percentage of the predicted in forced vital capacity (FVC), with stable percentages forced expiratory volume in the first second percentage (FEV1), resulting in a dysanaptic lung growth pattern [21, 22].

Clinical manifestations of our patients are consistent with the literature as tachypnoea, cough, wheezing, exercise intolerance, and hypoxaemia persisting for at least 6 weeks after bronchiolitis or pneumonia with respiratory insufficiency are the most common symptoms reported. Additionally, crackles, wheezing on auscultation, and hyperinflation are the most frequently reported signs [7]. In our cohort, crackles were prominent in the lower chest quadrants.

HRCT plays a pivotal role in the diagnosis of PIBO, with the mosaic perfusion pattern being considered distinctive when associated with a history of lower respiratory adenovirus infection [9]. Mosaic features and air trapping can be magnified by positioning the child in the lateral decubitus position [23]. All the remaining radiological features identified in our patients were consistent with the literature on PIBO, which reports, a prevalence of bronchiectasis of 96%, air trapping (92%), mosaic perfusion (88%), bronchial wall thickening (78%), atelectasis (66%) and mucus plugging (58%) and our patients are very similar to the literature in these terms. [24].

We would like to highlight that in the majority of our patients we observed bronchial and bronchiolar wall thickening, which can be regarded as a radiological marker indicating the persistence of an inflammatory process affecting bronchioles. This assertion finds support in a recent observational study, which identified patients with bronchial wall thickening as responsive to methylprednisolone pulses [25].

According to ERS recommendations we performed bronchoscopy in most cases, essentially to rule out or assess the presence of infective agents or other bronchial conditions especially dynamic ones such as bronchomalacia or other anatomical abnormalities. All our patients who underwent BAL fluid cytology showed a remarkably elevated neutrophilic count, which is likely the driving factor in the development of PIBO, as reported in the literature. Although, in all our cases, there was concurrent bacterial colonization, literature reports that neutrophilia is reported even without bacterial infection in PIBO [26, 27]. The excess of neutrophils persists in the sputum for several years [21]. Persistent airway inflammation is further documented by an increase in pro‐inflammatory cytokines such as IL‐1b, IL‐6, IL‐8, TNF‐a, and NFkB [27].

We performed biopsies in cases where a definitive diagnosis was not completely established after the CT scan and to help rule out other diseases in particular vasculitis. In our cohort, lung biopsies revealed variable pathological findings. Two patients with severe clinical presentations, who underwent VATS and thoracotomy respectively, showed narrowed, obliterated, and fibrotic bronchioles with predominantly lymphocytic inflammatory infiltrates, consistent with PIBO. Another patient had lymphocytic infiltrates in the airway wall and interstitium without bronchiolar narrowing or fibrosis. Conversely, two patients exhibited eosinophilic infiltrates in the interstitium and alveolar capillaries, but no bronchiolar structures were present in the samples. These three patients with less representative findings, along with one with inadequate sampling, underwent TBB. We considered a possible diagnosis of asthma in all of the patients especially in those who showed eosinophilic infiltrates. However, no patient presented with peripheral blood eosinophilia nor prick test sensitization nor clinical response to salbutamol.

It is important to note that obtaining representative samples with TBB is particularly challenging in young children due to the limited sample size and the patchy nature of airway involvement in PIBO, often leading to sampling errors. Despite lung biopsy being considered the gold standard for PIBO diagnosis, even with VATS or open lung biopsies, the variability in inflammation and focal airway involvement necessitates significant expertise in histological interpretation [7]. This highlights the need for less invasive diagnostic approaches.

Therefore, the diagnostic pathway increasingly relies on clinical evaluation and imaging criteria, such HRCT, to improve diagnostic accuracy while ruling out other chronic lung diseases, as recommended by the literature [28]. We would like to highlight that there are notable discrepancies in our health system and in health‐seeking behaviour by families compared to those in the South American population, which historically represents the best described population, making comparison hazardous. Future strategies, such as using transbronchial lung cryo‐biopsy, may provide more representative samples than traditional TBB with less morbidity compared to VATS or open lung biopsy in young children with PIBO.

The MBWSF_6_ offers crucial insights into ventilation inhomogeneity in children with postinfectious bronchiolitis obliterans (PIBO) [7]. At diagnosis, our cohort had a median lung clearance index (LCI 2.5%) of 14.5, significantly above the normal limit, indicating marked ventilation inhomogeneity. This aligns with the existing literature showing elevated LCI values correlate with lung air‐trapping on CT scans [28, 29]. The wide interquartile range (Q1: 10.5, Q3: 18.9) and minimum LCI of 10.4 highlight the variability in disease severity and lung function among patients. LCI measurement via multiple‐breath washout (MBW) is useful for diagnosing and monitoring PIBO, especially in young children who cannot perform conventional spirometry. Given the young age of most PIBO patients, LCI provides a noninvasive and sensitive method to detect early lung function abnormalities and monitor disease progression, making MBW a particularly useful tool in clinical management.

Dysphagia and chronic aspirations are known to contribute significantly to the progression of diffuse lung diseases. However, these aspects were scarcely investigated in our cohort, as no patient exhibited clinically evident dysphagia or imaging features indicative of chronic inhalation‐related lung damage. We administered methylprednisolone pulses in all patients because it appears to be the most effective treatment, especially if initiated early and if peri‐bronchitis is observed on imaging [25, 30].

Another treatment commonly used for BO is the triple therapy, known as the FAM regimen, which includes inhaled fluticasone, azithromycin, and montelukast. This regimen is sometimes utilized for PIBO as well. However, randomized trials evaluating its efficacy in BO post‐HSCT are still lacking, as only a phase 2 trial is available [31], and there are no reported data about FAM in PIBO. A similar approach was recently proposed called BAMA (budesonide, azithromycin, montelukast and acetylcysteine), but only a follow‐up study is available on this regimen, reporting improvements in nearly 70% of the cases [31, 32, 33]. We considered these therapeutic approaches as possible complementary treatments, and in most cases, we prescribed chronic treatment with azithromycin and inhaled corticosteroids. However, only one child received montelukast.

In two cases with a severe clinical course and subtle immunological abnormalities, we administered IVIG, following a treatment approach reported in a recent retrospective study involving 11 patients. This study showed a reduction in the number of hospitals visits due to infection, decreased frequency of hospitalizations, and discontinuation of oxygen therapy [34].

The decision to administer cyclophosphamide was based on its use in treatment regimens for refractory chILDs. This decision was made for a patient with Down syndrome who presented with progressive disease despite prior treatment with methylprednisolone pulse and IVIG [35, 36] Subsequently, this patient showed clinical and radiological improvement. Following this partial success, mycophenolate was initiated as a maintenance therapy [37, 38]. Except for these two cases who were exceedingly severe and thus required a higher treatment intensity, the remaining cases were remarkably homogenous in terms of clinical presentations, radiological characteristics, treatments received and clinical evolution. This represents a strength point compared to recently published studies with higher number of patients [39].

In our patients, physiotherapy was considered helpful by all the families, reporting improvements in secretion clearance, this aspect should be evaluated carefully as physiotherapy was started at the time of the first evaluation together or very soon after other interventions [40]. Moreover, there are no published data on physiotherapy effectiveness in PIBO [41].

## 5 Conclusions

In 2023, we saw an unprecedented rise in PIBO cases triggered by adenovirus‐associated lower respiratory infections. We attribute this surge to potential shifts in virus circulation due to COVID‐19 pandemic measures. We recommend a vigilant approach, especially if adenovirus is isolated during acute infection with a chronic respiratory cough. These cases should be considered as acute forms with predominant immunological activity, warranting aggressive methylprednisolone pulse therapy based on the patient's history and HRCT findings.

## Author Contributions

**A. Traunero:** investigation, writing – original draft, writing – review and editing, data curation. **S. Ghirardo:** writing – original draft, conceptualization, methodology, writing – review and editing, visualization. **M. Aldeco:** writing – review and editing, supervision, validation, data curation. **P. Pascolo:** data curation, validation, investigation. **S. Basilicata:** data curation, investigation. **L. Mazzari:** data curation, investigation. **M. Maschio:** supervision, validation. **A. Amaddeo:** supervision, writing – review and editing, visualization, project administration, methodology, formal analysis. **U. Krivec:** visualization, project administration, supervision, methodology, formal analysis.

## Ethics Statement

The study protocol was conducted in accordance to the General Authorization to Process Data for Scientific Research Purpose no. 9/2014, due to the retrospective nature of the study no dedicated signed consent was required.

## Conflicts of Interest

The authors declare no conflicts of interest.

## Supporting information

Table 1 Supporting material. Histopathological findings on lung biopsy, in brackets method to obtain lung biopsy sample.

## Data Availability

The data that support the findings of this study are available on request from the corresponding author. The data are not publicly available due to privacy or ethical restrictions.
