# Supplementary material for: Outbreak of Post‐Infectious Bronchiolitis Obliterans (PIBO) After Adenovirus Infection: A Case Series and Review of the Literature
Source: Pediatr Pulmonol. 2025 Apr 1;60(4):e71080. doi: 10.1002/ppul.71080 (PMC11960595; doi:10.1002/ppul.71080)
Supplement: Supplementary file 1 — Table 1 Supporting material. Histopathological findings on lung biopsy, in brackets method to obtain lung biopsy sample. [file PPUL-60-0-s001.docx]

| pts 1 | Preserved architecture with compression of the alveoli, moderately reactive pneumocytes, numerous eosinophils, and rare neutrophils in the interstitial space, along with macrophages present in the alveoli. No airway wall was found in the sample (TBB). |
| --- | --- |
| pts 4 | Bronchiolar fibrosis collapsed alveoli with lymphocytic infiltrate mainly CD4+ (T). |
| pts 7 | Atelectatic lung parenchyma; narrow bronchioles, with intense wall inflammatory infiltration, predominantly lymphocytes, and connective wall tissue proliferation in some of them. Some completely obliterated bronchioles, with connective tissue and mononuclear infiltrate in the lumen; hypercellular alveolar septa with mononuclear infiltrate and rare interstitial neutrophils; macrophages in the alveoli (VATS). |
| pts 8 | Eosinophils in the capillaries of alveolar septa are possibly indicative of tissue eosinophilia. No airway wall was found in the sample (TBB). |
| pts 9 | Intense predominantly lymphocytic inflammatory infiltrate in the lamina propria of the airway wall, mild edema; lymphocytic infiltrate and rare neutrophils in the alveolar septa (TBB). |
| pts 10 | Clot, inappropriate sample (TBB). |

Table 1 supplementary material. Histopathological findings on lung biopsy, in brackets method to obtain lung biopsy sample.

TBB=bronchoscopic transbronchial biopsy, T=thoracotomy, VATS=video thoracoscopic assisted surgery.
